# Increased cortical lesion load and intrathecal inflammation is associated with oligoclonal bands in multiple sclerosis patients: a combined CSF and MRI study

**DOI:** 10.1186/s12974-017-0812-y

**Published:** 2017-02-21

**Authors:** Gabriele Farina, Roberta Magliozzi, Marco Pitteri, Richard Reynolds, Stefania Rossi, Alberto Gajofatto, Maria Donata Benedetti, Francesco Facchiano, Salvatore Monaco, Massimiliano Calabrese

**Affiliations:** 1 Neurology B, Department of Neurological, Biomedical and Movement Sciences, University of Verona, Policlinico “G.B. Rossi” Borgo Roma, Piazzale L.A. Scuro, 10, 37134 Verona, Italy; 2 Unit of Neurology, Department of Clinical and Experimental Medicine, University of Sassari, Sassari, Italy; 3 Division of Brain Sciences, Faculty of Medicine, Imperial College London, London, UK; 4 Department EOMM, Istituto Superiore di Sanità, Rome, Italy

**Keywords:** CSF, MRI, Oligoclonal bands, IgG, Multiple sclerosis, OCB, Cytokines, Grey matter, Neuroinflammation, Neurodegeneration

## Abstract

**Background:**

Although IgG oligoclonal bands (OCBs) in the cerebrospinal fluid (CSF) are a frequent phenomenon in multiple sclerosis (MS) patients, their relationship with grey matter lesions, intrathecal/meningeal inflammation and clinical evolution has not been clarified yet.

The aim of our study was to assess the relationship between the OCBs, the inflammatory/neurodegenerative CSF profile at diagnosis, the cortical lesion load and the clinical evolution after 10 years.

**Methods:**

This is a 10-year observational, cross-sectional study based on a combined MRI, cognitive and CSF profiling of the examined patients.

Forty consecutive OCB-negative (OCB−) and 50 OCB-positive (OCB+) MS patients were included in this study. Both groups had mean disease duration of 10 years and were age and gender matched. Each patient underwent neurological and neuropsychological evaluation and 3-T MRI. Analysis of the presence and levels of 28 inflammatory mediators was performed in the CSF obtained from 10 OCB− MS, 11 OCB+ MS and 10 patients with other neurological conditions.

**Results:**

Increased number of CLs was found in OCB+ compared to OCB− patients (*p <* 0.0001), whereas no difference was found in white matter lesion (WML) load (*p =* 0.36). The occurrence of OCB was also associated with increased levels of neurofilament light chains and of several inflammatory mediators linked to B lymphocyte activity and lymphoid-neogenesis (CXCL13, CXCL12, CXCL10, TNFSF13, TNFSF13B, IL6, IL10) and other pro-inflammatory molecules, such as IFN-γ, TNF, MMP2, GM-CSF, osteopontin and sCD163. Finally, the occurrence of OCB was found associated with poor prognosis, from both physical and cognitive points of view.

**Conclusions:**

OCB at MS onset are associated with more severe GM pathology and with a more severe physical disability and cognitive impairment after 10 years. Increased levels of cytokines linked to B cell activation, lymphoid-neogenesis, and pro-inflammatory immune response in the CSF of OCB+ patients support the hypothesis of crucial role played by compartmentalized, intrathecal B cell response in the pathogenesis of CLs and OCB production.

**Electronic supplementary material:**

The online version of this article (doi:10.1186/s12974-017-0812-y) contains supplementary material, which is available to authorized users.

## Background

Multiple sclerosis (MS) is a chronic immune-mediated disease of the central nervous system (CNS) characterized by demyelination, neurodegeneration and axonal/glial pathology. Persistent intrathecal inflammation, demonstrated by the presence of oligoclonal bands (OCBs) in the cerebrospinal fluid (CSF) [1, 2], is one of the main hallmarks of MS in about 87.7% of MS patients [3]. The early detection of OCB in the disease course, together with the deposition of antibody, activation of complement and demyelination [4, 5], supports the role for B cells in MS pathogenesis. Clonally expanded populations of memory B and plasma cells have been found in the perivascular spaces, in the meningeal infiltrates, and in the CSF of patients with MS, even at the disease onset.

B cells express a specific chemokine receptor profile dependent upon their state of differentiation and external microenvironment. Several chemokines have been shown to influence CNS B cell trafficking and activity: CCL2, CCL3, CCL20, CXCL10, CXCL12 and CXCL13 [6]. Among these factors, increased CSF levels of CXCL13, one of the most important chemokine regulating B cell migration and differentiation in MS patients and EAE models [7–9], corroborate the hypothesis that the abnormal B cell immune response persists and becomes compartmentalized in the CNS. Diffuse inflammation, sometimes accompanied by the formation of ectopic tertiary lymphoid tissues (TLT), and recently detected in the inflamed meninges of post-mortem secondary progressive MS cases [10–12], may represent the niches within cerebral sulci in which the B cell response persists and plays its role in disease exacerbation. Inflammation within the subarachnoid space is suggested to be involved in the creation of an intrathecal inflammatory milieu that, through circulating CSF, baths the external adjacent cortical grey matter (GM) and mediates neuronal and glia pathological alterations [10, 11, 13]. As shown by several studies, the consequent GM damage is strictly correlated with the accumulation of physical and cognitive disability, and with the evolution of the progressive phase of the disease [14–17]. In a previous preliminary, cross-sectional study [18], we observed an association between cortical lesions (CLs) and OCB but no formal hypotheses about the origin of such association were suggested.

The aim of the present study was to confirm the relationship between OCB expression, cortical damage and the unfavourable clinical evolution and to test the hypothesis that the presence of OCB and CLs might be related to the intrathecal inflammation and to the presence of subarachnoid tertiary B cell lymphoid-like structures.

## Methods

### Patients

All consecutive OCB− patients having a diagnosis of MS according to the most recent MS diagnostic criteria [19] and who consecutively underwent neurological examination at the MS Centre of Verona University Hospital, between January and December 2014, were asked to participate in this study. Among these 44 patients, 2 were excluded since their diagnosis was not confirmed, 2 did not agree to participate, and 40 were enrolled in this cohort study. The mean disease duration for these patients was 10.5 ± 7.3 years. Among the 815 OCB+ patients who underwent neurological examination in our MS centre in the same period, an MS population sex, age and disease duration matched with OCB− population was randomly pulled out by a homemade algorithm (flow chart in Additional file 1). Steroid therapy during the 2 months before the enrolment was an exclusion criterion. See Table 1 for demographic and clinical characteristics of the two populations studied. Furthermore, 10 age- and gender-matched control patients, with other neurological conditions, have also been recruited. The study was approved by the local ethic committee, and informed consent was obtained from all patients.

**Table 1:** Clinical and MRI parameters at diagnosis and at the enrolment (whole population)

	OCB+	OCB−	Whole group	*p* value
*N* (f/m)	50 (34/16)	40 (28/12)	90 (62/28)	0.511
Age (years)	42.5 ± 10.4 (19–67)	42.4 ± 11.2 (17–69)	42.5 ± 10.7 (17–69)	0.943
Years from onset to diagnosis	3.0 ± 5.1 (0–21)	2.6 ± 3.9 (0–17)	2.8 ± 4.6 (0–21)	0.356
Years from diagnosis to enrolment	7.8 ± 5.2 (0–20)	7.9 ± 6.4 (0–22)	7.9 ± 5.7 (0–22)	0.918
Years from onset to enrolment (disease duration)	10.8 ± 7.0 (1–31)	10.5 ± 7.3 (1–29)	10.7 ± 7.1 (1–31)	0.878
EDSS at diagnosis	1.5 (0–3.0)	1.0 (0–3.5)	1.5 (0–3.5)	0.645
EDSS at enrolment	2.8 (1.0–8.0)	1.5 (0–6.5)	2.0 (1.0–8.0)	*<*0.0001
RRMS/SPMS at enrolment	35/15	36/4	71/19	0.018
Time to SPMS transition in months	123 ± 23 (98–187)	183 ± 13 (165–198)	136 ± 33 (98–198)	*<*0.0001
Cognitive impairment CN/mCI/sCI	30/9/11	34/5/1	64/14/12	0.013
CL number at enrolment	6.1 ± 6.1 (0–24)	2.2 ± 2.8 (0–11)	4.4 ± 5.3 (0–24)	*<*0.0001
WMLV at enrolment (cm^3^)	7.1 ± 3.6 (1.1–16.4)	6.5 ± 4.1 (1.3–18.1)	6.8 ± 4.0 (1.1–18.1)	0.258
Treatment until now				
None	0	5	5	0.033
Only 1° line^a^	34	28	62	
At least 2° line^a^	11	6	17	
At least 3° line^a^	5	1	6	

*EDSS* Expanded Disability Status Scale, *RRMS* relapsing-remitting multiple sclerosis, *SPMS* secondary progressive multiple sclerosis, *CN* cognitively normal, *mCI* mild cognitive impairment, *sCI* severe cognitive impairment, *CL* cortical lesion, *WML* white matter lesion

Data are reported as mean ± standard deviation (range). For the EDSS, median and (range) are provided

^a^1° line drugs: IFN-beta, GA, azathioprine; 2° line drugs: fingolimod, natalizumab; 3° line drug: cyclophosphamide, mitoxantrone

### Clinical information

Each patient was assessed by means of the Expanded Disability Status Scale (EDSS) [20] at the time of inclusion in the study. Previous EDSS scores were retrospectively acquired from the clinical records of each patient. Data about previous and actual disease-modifying therapies were collected. Patients were classified into four categories, according to the type of drug administered (Table 1).

### Neuropsychological assessment

Neuropsychological assessment was performed at the time of enrolment in the present study by means of the brief repeatable battery (BRB) of neuropsychological tests [21]—except the PASAT-2 subtest. The BRB is composed by tests of verbal learning and delayed memory recall (Selective Reminding Test, SRT), visuo-spatial learning and delayed memory recall (10/36 Spatial Recall Test, SPART), auditory information processing speed, working memory, attention, and calculation (Paced Auditory Serial Addition Test, PASAT), visual information processing speed and attention (Symbol Digit Modalities Test, SDMT), and semantic verbal fluency (Word List Generation, WLG). Test scores were considered failed according to the cut-off scores (5th percentile) derived from the Italian normative data [21]. Previous neuropsychological evaluations were excluded from the analysis since the types of tests administered were heterogeneous and then not comparable.

The presence of depression was assessed by means of the Beck Depression Inventory second edition (BDI-II) [22].

MS patients were classified into three groups: cognitively normal (CN = no failed test of the BRB), mild cognitive impairment (mCI = up to two failed tests), and severe cognitive impairment (sCI = three or more failed tests).

### CSF analysis

The CSF, collected at the time of diagnosis from all the examined MS patients for diagnostic purposes, was then available for protein analysis from 21 (11 OCB+ and 10 OCB−, strictly representative of the two groups of examined MS patients) out of the 90 MS patients enrolled in the study and from 10 control patients (affected by other inflammatory neurological diseases, OIND). CSF sample collection and preparation were performed more than 2 months after the last relapse according to in-house guidelines (used since 1994 by the bio-bank at the MS Centre of Verona), which were in line with the Consensus Guidelines for CSF and Blood Biobanking [23]. CSF, obtained at the disease diagnosis, was centrifuged soon after collection, and both the supernatant fraction and the cell pellet were separately stored at −80 °C until use. The presence of OCBs was performed by using iso-electric focusing method and blindly assessed by two independent examiners [24]. When possible, the assessment of the OCB in OCB− MS patients was re-tested after second lumbar puncture showing confirmation of OCB absence.

The presence and levels of 28 inflammatory mediators, including either the major pro-inflammatory mediators or molecules mainly related to the B cell immune response (Table 2), were assessed using a combination of immune-assay multiplex techniques based on the Luminex technology (Table 2; Bio-Plex X200 System equipped with a magnetic workstation, BioRad, Hercules, CA, USA). The CSF analysis was optimized and performed by two independent investigators (RM and SR), blinded with respect to the presence/absence of OCB and to the clinical characteristics of the patient and according to previously published procedures [25, 26]. All samples were run in duplicate and a number of molecules have been measured in different immune-assay platform in order to verify the reproducibility and consistence of the results. The CSF level of each protein detected during the analysis was normalized to the protein concentration of each CSF sample (measured by Bradford protocol).

**Table 2:** Methodological details of the examined inflammatory proteins in the CSF of MS patients by Bio-Plex immunoassay System

		Detection limit (pg/ml)	Intra-assay variation (%)	Recovery percentage range (90–110%)
BCA1/CXCL13	Chemokine (C-X-C motif) ligand 13 (CXCL13) or B lymphocyte chemoattractant (BLC) or B cell-attracting chemokine 1 (BCA-1)	0.42–6694	6.0	100
SDF1αβ/CXCL12	C-X-C motif chemokine 12 (CXCL12) or stromal cell-derived factor 1 (SDF1)	12.73–160,858	5.4	104
6Ckine/CCL21	Chemokine (C-C motif) ligand 21	11.30–160,686	4.0	104
IP10/CXCL10	C-X-C motif chemokine 10 (CXCL10) or interferon gamma-induced protein 10 (IP-10)	1.04–14,668	1.5	95
BAFF/TNFSF13B	B cell activating factor (BAFF) or tumour necrosis factor ligand superfamily member 13B (TNFSF13B)	70.19–197,936	6.3	100
APRIL/TNFSF13	A proliferation-inducing ligand (APRIL), or tumour necrosis factor ligand superfamily member 13 (TNFSF13)	906.31–4,745,900	7.5	103
TWEAK/TNFSF12	TNF-related weak inducer of apoptosis (TWEAK) or tumour necrosis factor ligand superfamily member 12	3.59–6346	4.4	106
LIGTH/TNFSF14	Tumour necrosis factor superfamily member 14 (TNFSF14)	1.92–4647	5.8	98
TNFα	Tumour necrosis factor alpha	0.24–13,148	2.5	99
sTNFR1	Soluble- tumour necrosis factor-receptor 1	2.33–48,047	5.0	100
sTNFR2	Soluble- tumour necrosis factor-receptor 2	3.72–63,484	4.2	98
MIP3β/CCL19	Chemokine (C-C motif) ligand 19 (CCL19) or EBI1 ligand chemokine (ELC) or macrophage inflammatory protein-3-beta (MIP-3-beta)	3.08–49,150	2.8	103
MIG/CXCL9	Chemokine (C-X-C motif) ligand 9 (CXCL9) or monokine induced by gamma interferon (MIG)	1.19–23,643	5.4	97
sCD163	Soluble-CD163 (Cluster of Differentiation 163)	0.38–10,497	6.0	102
GM-CSF	Granulocyte-macrophage colony-stimulating factor (GM-CSF) or colony-stimulating factor 2 (CSF2)	2.32–34,028	5.2	104
INFα2	Interferon alpha-2	1.24–19,334	5.3	99
INFβ	Interferon beta	1.50–8727	6.0	100
INFγ	Interferon gamma	1.50–2326	3.0	99
IL1β	Interleukin-1 beta	0.15–2326	2.0	102
IL2	Interleukin-2	0.47–10,334	4.0	98
IL4	Interleukin-4	0.35–5258	6.0	100
IL6	Interleukin-6	0.68–57,162	3.0	99
IL8/CXCL8	Interleukin-8 (C-X-C motif) or chemokine ligand 8, CXCL8)	11.56–8523	1.0	103
IL10	Interleukin-10	0.84–2997	3.0	98
OPN	Osteopontin	123.64–286,066	7.3	100
MMP1	Matrix metallopeptidase 1	298.2–216,430	4.5	100
MMP2	Matrix metallopeptidase 2	90.63–2,109,030	3.3	104
MIF	Macrophage migration inhibitor factor	8.25–456,461	2.6	103

The levels of neurofilament light chain (NF-L) in CSF were measured using the Human NF-light ELISA kit (MyBioSource, San Diego, CA, USA) according to the manufacturer’s instructions. The monoclonal antibody was allowed to bind to the antigen for 90 min at 37 °C and the unbound sample was washed away followed by addition of a biotinylated human NF-L antibody at 37 °C for 1 h. The enzyme-conjugate liquid was added after plate washed for 30 min at 37 °C and plate was then washed. The plate was added with 3,3′,5,5′-tetrametilbenzidine (TMB) substrate according to the manufacturer’s instructions and read at λ450 nm within 10 min and the quantification was carried out on VICTORTM X3 2030 Multilabel Plate Reader (Perkin Elmer, Walluf, Germany). Intra-assay variability (coefficients of variation) samples were below 10%. Data obtained were normalized for total protein concentration (mg/ml).

### Image acquisition protocol

Each patient was scanned at enrolment using a 3.0-T Philips Achieva MRI (Philips Medical Systems, Best, The Netherlands), acquiring the following sequences:

- 3D fluid attenuated inversion recovery (FLAIR) TR/TE = 5500/292 ms, TI = 1650 ms, voxel dimension of 1 × 1 × 1 mm
- 3D double inversion recovery (DIR) TR/TE = 5500/292 ms, TI1/TI2 = 525 ms/2530 ms voxel dimension of 1 × 1 × 1 mm
- 3D T1 weighted fast field echo (FFE) TR/TE = 8.4/3.7 ms, voxel dimension of 1 × 1 × 1 mm

Previous MRI examinations acquired at the time of diagnosis (i.e. within 1 month from CSF collection) using a 1.5-T Philips Achieva MRI scanner, were available for 35 OCB− and 48 OCB+ patients.

The following sets of images were acquired at a 1.5-T scanner:

- 3D DIR: TR = 15,631 ms, TE = 25 ms, TI = 3400 ms, delay = 325 ms, ETL = 17, 50 contiguous axial slices with a thickness = 3 mm, matrix size = 130 × 256, and field of view (FOV) = 250 × 200 mm
- 3D FLAIR: TR = 10,000 ms, TE = 120 ms, TI = 2500 ms, ETL = 23, 50 contiguous axial slices with a thickness = 3.0 mm, matrix size = 172 × 288, and FOV = 250 × 200 mm
- 3D FFE: 120 contiguous axial slices with the off-centre positioned on zero, TR = 25 ms, TE = 4.6 ms, flip angle = 30°, slice thickness = 1.2 mm, matrix size = 256 × 256, and FOV = 250 × 250 mm

No major hardware upgrades were carried out on the MRI scanners during the study.

### Image analysis

All DIR images were assessed by consensus of two experienced observers (MC and GF) blinded to patients’ identities and clinical data. Each CL was identified according to the recent recommendations for CL scoring in patients with MS [27]. All FLAIR images were assessed by consensus of the same experienced observers, and the white matter lesion load was quantified. On FLAIR images, white matter lesion volume was quantified after lesion identification using a semi-automated local thresholding technique based on Fuzzy C-mean algorithm, part of the Medical Images Processing Analysis and Visualization (MIPAV) software (http://mipav.cit.nih.gov).

**Fig. 1:**
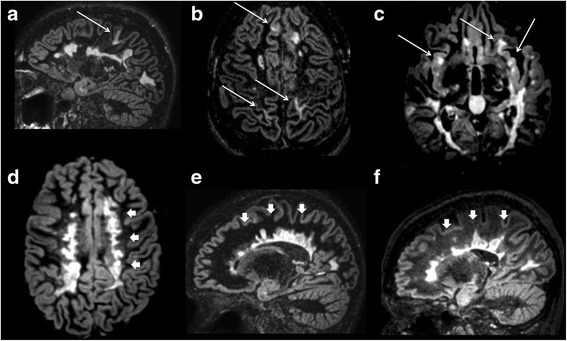
3D double inversion recovery images of three RRMS OCB+ patients (**a**–**c**) and three RRMS OCB− patients (**d**–**f**). OCB+ patients show both white and grey matter demyelination (*arrows*) including insular lesions (*arrows*, **c**). OCB− patients do not show any grey matter lesion despite the severe white matter demyelination especially in the periventricular region (*arrowheads*, **d**–**f**)

### Statistics

Since EDSS scores were not normally distributed, the Wilcoxon or Mann-Whitney test was used to compare the two MS populations with respect to their EDSS score at diagnosis and at enrolment.

Pearson chi-square was used to test the difference between the two MS groups in terms of categorical data (i.e., female/male ratio; disease form: relapsing-remitting MS vs secondary progressive MS) and cognitive status (a ranked scale: CN vs mCI vs sCI). Univariate correlations among continuous variables were assessed using the Pearson correlation coefficient and those among discrete variables with the Spearman rank correlation coefficient. Differences among groups in continuous variables as age, time from diagnosis to enrolment, time from onset to diagnosis, and lesion load were assessed through analysis of variance (ANOVA). Mann-Whitney tests were also used to test differences in terms of CSF protein levels between OCB+ and OCB− MS patients. KEGG pathway analysis was also used to perform unsupervised pathway analysis on the CSF inflammatory mediators levels. The Bonferroni correction was applied. All statistical analyses were performed using SPSS v. 21 and R (http://www.r-project.org).

## Results

### Clinical parameters

Despite the same mean age, disease duration, and EDSS at the time of diagnosis (see Table 1), the OCB+ group showed more severe disease evolution. Ten years after the disease onset, the median EDSS score of OCB+ patients (2.8 [1.0–8.0]) was significantly higher than that of OCB− patients (1.5 [0–6.5], *p <* 0.0001). The proportion of patients who entered the SP phase was higher in OCB+ (15/50, 30.0%) than in OCB− (4/40, 10.0%, *p =* 0.018) and the time to reach the progressive phase was shorter in OCB+ (mean 123 ± 23 months; range 98–187 months) than in OCB− (mean 183 ± 13 months; range 165–198 months; *p <* 0.0001) patients. The greater severity of the pathological process in the OCB+ patients was also confirmed by the need of administering a higher rate of second- and third-line drug treatments compared to OCB− patients (*p =* 0.033).

Finally, a significant difference was also observed between OCB+ and OCB− patients according to their classification in the three groups considering the number of failed neuropsychological tests (*p =* 0.013): specifically, 30 OCB+ patients (60%) were classified as CN, 9 (18%) were classified as mCI, and 11 (22%) were classified as sCI; on the contrary, 34 OCB− patients (85%) were classified as CN, 5 (12.5%) were classified as mCI, and only 1 (2.5%) was classified as sCI (Table 1).

### Imaging data

At the time of diagnosis, OCB+ patients showed 61.1% higher number of CLs (1.8 ± 2.0 [0–5]) compared to OCB− patients (1.1 ± 1.7 [0–6]; *p =* 0.041). CLs were detected in 25/48 (52%) OCB+ and in 11/35 (31%) OCB− patients (*p =* 0.075). No significant difference was observed in terms of WM lesion load (*p =* 0.465) between the OCB+ (mean = 2.0 ± 0.9 cm^3^, range = 0.5–4.4 cm^3^) and OCB− (mean = 1.6 ± 2.0 cm^3^, range = 1.0–5.9 cm^3^) patients.

After 10 years of disease, OCB+ patients showed 2.7 times higher number of CLs (6.1 ± 6.05 [0–24]) compared to OCB− patients (2.2 ± 2.8 [0–11]; *p <* 0.0001). CLs were detected in 46/50 (92%) of OCB+ and in 23/40 (57%) of OCB− patients (*p <* 0.0001) Fig. 1.

As detected at the time of diagnosis, after 10 years no significant difference was observed in terms of WM lesion load (*p =* 0.258) between the OCB+ (mean = 7.1 ± 3.6 cm^3^, range = 1.1–16.4 cm^3^) and OCB− (mean = 6.5 ± 4.1 cm^3^, range = 1.3–18.1 cm^3^) patients.

### CSF analysis (at the time of diagnosis)

Among the clinical CSF parameters, significant increased in CSF cell count was found in OCB+ patients compared to controls (*p =* 0.02); furthermore, increased IgG index was detected in the CSF of OCB+ group compared to both controls (*p =* 0.0075) and to the OCB− group (*p =* 0.039).

**Fig. 2:**
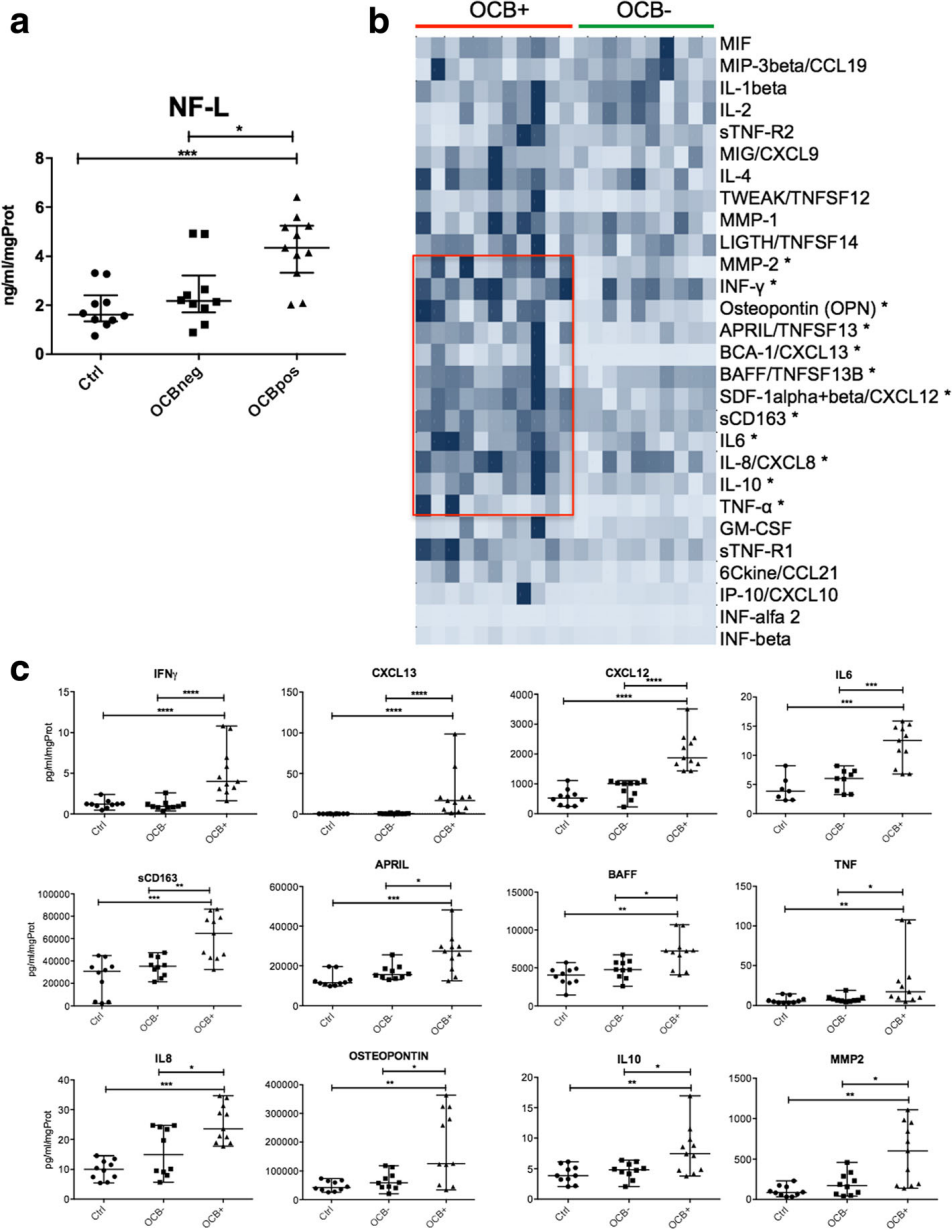
Protein analysis of the presence and levels of neurofilament light chains (NF-L) and inflammatory mediators in CSF. **a** NF-L protein levels have been analysed in the CSF of controls, OCB− and OCB+ MS patients by using ELISA assessment, showing significant increased levels in OCB+ MS patients compared to both controls and OCB− MS patients. **b** Cluster analysis showing the level of expression of the 28 inflammatory mediators examined in OCB− and OCB+ MS patients: the red rectangle outlines a cluster of molecules significantly overexpressed only in OCB+ MS patients. **c** Statistical representation of the presence and levels of inflammatory mediators significantly overexpressed in OCB+ compared to controls and to OCB− MS patients. (Statistical analysis performed by using Mann-Whitney test; *p* value *<0.05; **<0.01; ***<0.001)

Significantly higher levels of NF-L were detected in the CSF of OCB+ patients compared to both controls (fold change = 2.260; *p =* 0.007) and OCB− patients (fold change = 2.165; *p =* 0.024) (Fig. 2a). Among the 28 inflammatory molecules examined, 12 were significantly overexpressed (*p <* 0.05) in OCB+ compared to both controls and OCB− MS patients (Fig. 2b, c): IL6, IL8, IL10, CXCL13, CXCL12, TNF, APRIL, BAFF, IFNγ, MMP2, osteopontin (OPN), sCD163. No difference in the CSF levels of these molecules has been revealed in the OCB− group compared to controls. Although a trend towards an increase of few molecules was observed, such as MIF and CCL19, no significant overexpression was found in the CSF of OCB− patients. By using unsupervised pathway analysis, the occurrence of OCB in the CSF at MS onset was associated with the overexpression of molecules related to the B cell immune response, such as the lymphoid chemokines CXCL13 (*p <* 0.001), CXCL12 (*p <* 0.001), factors related to B cell activation and differentiation such as BAFF (*p =* 0.019), APRIL (*p =* 0.015), IL6 (*p =* 0.0005), and IL10 (*p =* 0.035) (Fig. 2b) or to those with a B cell regulatory role, such as OPN (*p =* 0.019). Concomitant overexpression of the major pro-inflammatory molecules, such as IFNγ (*p <* 0.001), TNF (*p =* 0.012), IL8 (*p =* 0.042), MMP2 (*p =* 0.015) and the marker of activated monocytes, sCD163 (*p =* 0.003), were found to be associated with the presence of OCB (Fig. 2b, c).

**Fig. 3:**
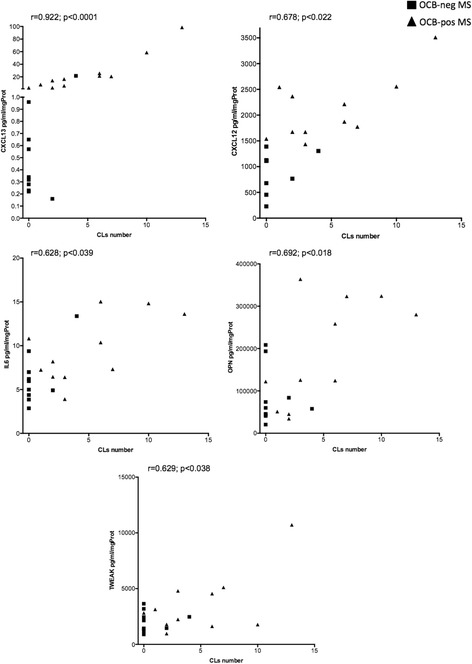
Pearson correlation analysis between the CSF levels of overexpressed proteins and the number of cortical lesions detected by 3 T MRI in the subgroup of the 21 MS patients. All the represented correlations involve molecules related to B cell chemo-attraction (CXCL13, CXCL12) and activity (IL6, OPN, TWEAK). The *r* and *p* values are reported for each examined correlation in the correspondent graph

**Fig 4:**
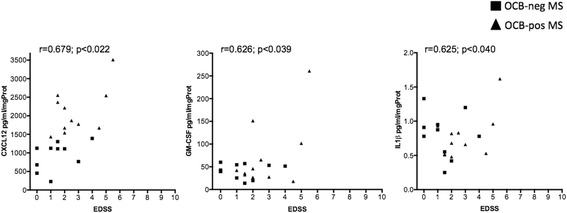
Pearson correlation analysis between the CSF levels of overexpressed proteins and the EDSS values of the subgroup of the 21 MS patients at the follow-up. The *r* and *p* values are reported for each examined correlation in the correspondent graph

In the OCB+ MS patients, CL load significantly correlated with levels of several molecules linked to the B cell immune response, such as CXCL13 (*r =* 0.922; *p <* 0.001), CXCL12 (*r =* 0.678; *p =* 0.022), OPN (*r =* 0.692; *p =* 0.018), IL6 (*r =* 0.628; *p =* 0.039), TWEAK (*r =* 0.629; *p =* 0.038) (Fig. 3). EDSS scores positively correlated with the protein levels of CXCL12 (*r =* 0.679; *p =* 0.022), GM-CSF (*r =* 0.626; *p =* 0.039) and IL1beta (*r =* 0.625; *p =* 0.040) (Fig. 4).

## Discussion

In this study we showed that a more inflammatory intrathecal profile and a more severe cortical demyelination are usually associated to the occurrence of OCB at MS diagnosis and to a more severe clinical evolution after 10 years of follow-up. To draw this conclusion, we evaluated the evolution of physical and cognitive disability, the time to progressive phase of the disease and the number of CLs in relapsing-remitting multiple sclerosis (RRMS) patients with and without OCB, at diagnosis and after about 10 years of disease duration. From the clinical point of view, physical and cognitive results suggest that the presence of OCB at diagnosis can be associated with a worse prognosis. In addition the presence of OCB in the CSF of MS patients is linked to increased CSF cell counts and IgG index, that could reflect the higher inflammatory condition of the CSF of the OCB+ group. In particular, while the cell count significantly differs in OCB+ patients only compared to controls, the significant increase in IgG levels in OCB+ compared to both controls and OCB− may demonstrate the preponderance of the humoral response in OCB+ MS patients. In OCB+ group, the number of patients who entered the progressive phase of the disease was significantly higher, and the time to reach the progressive phase was significantly shorter than in OCB− patients group. Such differences of disease course could be explained by the presence of higher intrathecal inflammation and by the consequent higher cortical damage. Indeed, although the presence of CLs was not exclusive of patients with OCB, we showed that patients with OCB, despite the similar age, disease duration and WM lesion load, had a substantial increase of the CL load compared to patients without OCB, already at the time of diagnosis. Our hypothesis is that the presence of OCB might be an epiphenomenon of the compartmentalized intrathecal (meningeal) B cell response, which has been suggested to be associated with increased subpial demyelination and neuronal damage, both hallmarks of more rapidly progressive disease [12, 28–31]. While no difference in the levels of B cell-related CSF inflammatory mediators was found between controls and OCB− patients, it can be not excluded that differences in others CSF mediators may occur.

To provide more supporting evidence, we assessed the intrathecal CSF profile of a subgroup of patients with and without OCB. Our results confirmed that OCB+ patients have a greater intrathecal inflammatory activity already at the diagnosis. Such intrathecal inflammation was characterized by overexpression of molecules related to B cell differentiation/activity, inflammatory immune response and monocyte activation compared to both control and OCB− groups. Indeed, high levels of lymphoid chemokines CXCL13 and CXCL12, having a key role in the regulation of B cell migration and compartmentalization within secondary lymphoid organs [9], IL6 and IL10, involved in plasma blast differentiation/class switching as well as B cell regulatory immune activity, and BAFF, APRIL, and osteopontin, involved in the balance between activation, survival and apoptosis of B and T cells [30], were observed in OCB+ MS patients only. Our results confirm and extend previous observations of high levels of CXCL13 and increased numbers of naïve and memory B cells expressing its receptor, CXCR5, in the CSF of MS patients with intrathecal IgG synthesis and characterized by a more aggressive disease course [31–35]. Moreover, by stratifying patients on the basis of the OCB presence at diagnosis, we observed a direct correlation between high levels of CXCL13 (and other B cell-related molecules), CL load, and both long-term physical and cognitive disability. From the neurodegenerative point of view, the elevated level of CXCL12 detected in OCB+ MS patients, together with the high levels of NF-L discovered in the same MS patients, is in line with the high level of GM damage as suggested by the high number of CLs observed in OCB+ MS patients, suggesting a higher degree of neuronal and axonal damage in this subgroup of patients [36]. Indeed, in addition to its role in the immune system, CXCL12, which is produced by activated astrocytes, can induce neuronal apoptosis in certain conditions and promote survival of neurons in others [37]. Metalloproteinase (MMP)-2, that we also found overexpressed in the OCB+ MS patients, could have a direct role in cortical tissue damage by breakdown of extracellular matrix but could be also able to convert CXCL12 into a neurotoxic form [32].

The increased expression of several inflammatory molecules such as IFNγ, TNF, IL8, IL10, IL6, MMP2, and osteopontin found in OCB+ MS patients indicate higher levels of intrathecal inflammatory activity that may be directly involved in the cortical tissue damage, as well as indirectly by activation of resident glial cells. In particular, the high levels of IFNγ and TNF in the CSF of OCB+ MS patients with higher CL load is in agreement with the finding of increased IFNγ and TNF gene and protein expression in paired post-mortem meninges and CSF obtained from secondary progressive multiple sclerosis (SPMS) cases with increased meningeal inflammation, cortical damage and rapid disease progression [38]. Finally, the concurrent finding in OCB+ MS patients of increased levels of sCD163, that was proposed as possible informative marker of activated macrophages and disease activity [39–45], might suggest that an elevated intracerebral lymphocytic and macrophage activity is linked to more severe CL load and disease severity.

The intrathecal B cell activity and severe cortical pathology observed in OCB+ MS patients is also consistent with our previous studies demonstrating the presence of abnormal inflammation and tertiary lymphoid-like organs, rich in B-lymphocytes and plasma cells, in the meningeal infiltrates in the subarachnoid space in SPMS and primary progressive MS cases with a more rapid and severe disease progression [10, 12, 46]. Such a relationship would confirm our hypothesis that meningeal immune cell infiltrates may represent niches for the perpetuation of plasma cell activity and, therefore, together with the presence of long-lived plasma cells, of production sites of OCB immunoglobulins and of inflammatory factors. These inflammatory factors may diffuse across the glia-limitans and play a crucial role in the subpial damage, inducing demyelination, neurodegeneration, and glia alteration mediating a more severe and rapid progression [13, 38]. These events could occur since the early phases of the disease as suggested by the higher number of CLs observed in OCB+ patients since disease diagnosis and by other studies demonstrating the presence of increased meningeal inflammation [47] and meningeal enhancement [48] associated with increase subpial cortical demyelination in a subgroup of MS patients characterized by early and rapid disease progression.

We are aware that our study is not without limitations: first of all the limited number of the patients whose CSF was examined does not allow to drive any definitive conclusion; in addition, the limited number of patients does not allow further sub-analyses, stratifying the patients according to other clinical parameters (e.g., comparison between RRMS and SPMS forms of the OCB+ and OCB− groups, comparison of the same treatment lines, comparison of different MS forms in the same OCB group).

Moreover, although our MRI protocol was performed at a 3-T scanner and included a double inversion recovery sequence, a significant proportion of CLs could have escaped our detection. Finally, the retrospective nature of the study could be considered a limitation. However, it allowed us to choose patients whose diagnosis was confirmed by a very long clinical and radiological observation, and permitted us to correlate, for the first time, long-term clinical and radiological outcomes with the inflammatory features of the CSF obtained at the diagnosis.

## Conclusions

The present study confirms and extends our previous observation [18] of strong relationship between OCB and CLs in MS and shows that after almost 10 years of disease duration the number of CLs was about threefold higher in OCB+ compared to OCB− patients, despite the same WM lesion load both at diagnosis and at follow-up. Intrathecal inflammation, in particular linked to B cell immune response, seems to be involved in the production of OCB and in the pathogenesis of cortical damage in MS. Although our data needs to be confirmed in different and larger MS cohorts and by longer perspective follow-up, we suggest that a well-defined pro-inflammatory CSF profile is associated with early occurrence of OCB in the CSF, with development of a more severe cortical pathology, and with a worse clinical (physical and cognitive) prognosis. These results indicate that the evaluation of intrathecal inflammation, combined with the assessment of GM lesion load at the time of diagnosis, might constitute a crucial prognostic tool, which could be helpful in selecting the most appropriate therapy.

## Additional file

Additional file 1: Diagram of the studied populations. (PPTX 61 kb)

## Abbreviations

BRB: Brief repeatable battery
CL: Cortical lesion
CN: Cognitively normal
CNS: Central nervous system
CSF: Cerebrospinal fluid
DIR: Double inversion recovery
EDSS: Expanded Disability Status Scale
FLAIR: Fluid attenuated inversion recovery
GM: Grey matter
IgG: Immunoglobulin G
mCI: Mild cognitive impairment
MRI: Magnetic resonance imaging
MS: Multiple sclerosis
NF-L: Neurofilament light chains
OCB−: Oligoclonal bands negative
OCB+: Oligoclonal bands positive
OIND: Other inflammatory neurological diseases
PASAT: Paced Auditory Serial Addition Test
RRMS: Relapsing-remitting multiple sclerosis
sCI: Severe cognitive impairment
SPMS: Secondary progressive multiple sclerosis
WM: White matter
WML: White matter lesion

## References

[1] Kabat EA, Moore DH, Landow H (1942). An electrophoretic study of the protein components in cerebrospinal fluid and their relationship to the serum proteins. J Clin Invest.

[2] Villar LM, Masterman T, Casanova B, Gómez-Rial J, Espiño M, Sádaba MC (2009). CSF oligoclonal band patterns reveal disease heterogeneity in multiple sclerosis. J Neuroimmunol.

[3] Dobson R, Ramagopalan S, Davis A, Giovannoni G (2013). Cerebrospinal fluid oligoclonal bands in multiple sclerosis and clinically isolated syndromes: a meta-analysis of prevalence, prognosis and effect of latitude. J Neurol Neurosurg Psychiatry.

[4] Meinl E, Krumbholz M, Hohlfeld R (2006). B lineage cells in the inflammatory central nervous system environment: migration, maintenance, local antibody production, and therapeutic modulation. Ann Neurol.

[5] Kanter JL, Narayana S, Ho PP, Catz I, Warren KG, Sobel RA (2006). Lipid microarrays identify key mediators of autoimmune brain inflammation. Nat Med.

[6] Blauth K, Owens GP, Bennett JL (2015). The Ins and outs of B cells in multiple sclerosis. Front Immunol.

[7] Smith JR, Braziel RM, Paoletti S, Lipp M, Uguccioni M, Rosenbaum JT (2003). Expression of B-cell-attracting chemokine 1 (CXCL13) by malignant lymphocytes and vascular endothelium in primary central nervous system lymphoma. Blood.

[8] Magliozzi R, Columba-Cabezas S, Serafini B, Aloisi F (2004). Intracerebral expression of CXCL13 and BAFF is accompanied by formation of lymphoid follicle-like structures in the meninges of mice with relapsing experimental autoimmune encephalomyelitis. J Neuroimmunol.

[9] Cyster JG (2005). Chemokines, sphingosine-1-phosphate, and cell migration in secondary lymphoid organs. Annu Rev Immunol.

[10] Magliozzi R, Howell O, Vora A, Serafini B, Nicholas R, Puopolo M (2007). Meningeal B-cell follicles in secondary progressive multiple sclerosis associate with early onset of disease and severe cortical pathology. Brain.

[11] Magliozzi R, Howell OW, Reeves C, Roncaroli F, Nicholas R, Serafini B (2010). A gradient of neuronal loss and meningeal inflammation in multiple sclerosis. Ann Neurol.

[12] Howell OW, Reeves CA, Nicholas R, Carassiti D, Radotra B, Gentleman SM (2011). Meningeal inflammation is widespread and linked to cortical pathology in multiple sclerosis. Brain.

[13] Calabrese M, Magliozzi R, Ciccarelli O, Geurts JJG, Reynolds R, Martin R (2015). Exploring the origins of grey matter damage in multiple sclerosis. Nat Rev Neurosci.

[14] Rovaris M, Filippi M (2000). MRI correlates of cognitive dysfunction in multiple sclerosis patients. J Neurovirol.

[15] Calabrese M, Poretto V, Favaretto A, Alessio S, Bernardi V, Romualdi C (2012). Cortical lesion load associates with progression of disability in multiple sclerosis. Brain.

[16] Filippi M, Preziosa P, Copetti M, Riccitelli G, Horsfield MA, Martinelli V (2013). Gray matter damage predicts the accumulation of disability 13 years later in MS. Neurology.

[17] Zivadinov R, Uher T, Hagemeier J, Vaneckova M, Ramasamy DP, Tyblova M, et al. A serial 10-year follow-up study of brain atrophy and disability progression in RRMS patients. Mult Scler. 2016;22(13):1709–18. 10.1177/1352458516629769 26883943

[18] Calabrese M, De Stefano N, Atzori M, Bernardi V, Mattisi I, Barachino L (2007). Detection of cortical inflammatory lesions by double inversion recovery magnetic resonance imaging in patients with multiple sclerosis. Arch Neurol.

[19] Polman CH, Reingold SC, Banwell B, Clanet M, Cohen JA, Filippi M (2011). Diagnostic criteria for multiple sclerosis: 2010 revisions to the McDonald criteria. Ann Neurol.

[20] Kurtzke JF (1983). Rating neurologic impairment in multiple sclerosis: an expanded disability status scale (EDSS). Neurology.

[21] Amato MP, Portaccio E, Goretti B, Zipoli V, Ricchiuti L, De Caro MF (2006). The Rao’s Brief Repeatable Battery and Stroop Test: normative values with age, education and gender corrections in an Italian population. Mult Scler.

[22] Beck AT, Steer RA, Brown GK (1996). Manual for the Beck depression inventory-II.

[23] Teunissen CE, Tumani H, Bennett JL, Berven FS, Brundin L, Comabella M (2011). Consensus guidelines for CSF and blood biobanking for CNS biomarker studies. Mult Scler Int.

[24] Freedman MS, Thompson EJ, Deisenhammer F, Giovannoni G, Grimsley G, Keir G (2005). Recommended standard of cerebrospinal fluid analysis in the diagnosis of multiple sclerosis: a consensus statement. Arch Neurol.

[25] Tanaka M, Matsushita T, Tateishi T, Ochi H, Kawano Y, Mei F-J (2008). Distinct CSF cytokine/chemokine profiles in atopic myelitis and other causes of myelitis. Neurology.

[26] Studer V, Rossi S, Motta C, Buttari F, Centonze D (2014). Peripheral B cell depletion and central proinflammatory cytokine reduction following repeated intrathecal administration of rituximab in progressive Multiple Sclerosis. J Neuroimmunol.

[27] Geurts JJG, Roosendaal SD, Calabrese M, Ciccarelli O, Agosta F, Chard DT (2011). Consensus recommendations for MS cortical lesion scoring using double inversion recovery MRI. Neurology.

[28] Haider L, Zrzavy T, Hametner S, Höftberger R, Bagnato F, Grabner G (2016). The topograpy of demyelination and neurodegeneration in the multiple sclerosis brain. Brain.

[29] Serafini B, Rosicarelli B, Magliozzi R, Stigliano E, Aloisi F (2004). Detection of ectopic B-cell follicles with germinal centers in the meninges of patients with secondary progressive multiple sclerosis. Brain Pathol.

[30] Ma N, He Y, Xiao H, Han G, Chen G, Wang Y (2014). BAFF maintains T-cell survival by inducing OPN expression in B cells. Mol Immunol.

[31] Krumbholz M, Theil D, Cepok S, Hemmer B, Kivisäkk P, Ransohoff RM (2006). Chemokines in multiple sclerosis: CXCL12 and CXCL13 up-regulation is differentially linked to CNS immune cell recruitment. Brain.

[32] Sellebjerg F, Börnsen L, Khademi M, Krakauer M, Olsson T, Frederiksen JL (2009). Increased cerebrospinal fluid concentrations of the chemokine CXCL13 in active MS. Neurology.

[33] Haas J, Bekeredjian-Ding I, Milkova M, Balint B, Schwarz A, Korporal M (2011). B cells undergo unique compartmentalized redistribution in multiple sclerosis. J Autoimmun.

[34] Ferraro D, Galli V, Vitetta F, Simone AM, Bedin R, Del Giovane C (2015). Cerebrospinal fluid CXCL13 in clinically isolated syndrome patients: association with oligoclonal IgM bands and prediction of multiple sclerosis diagnosis. J Neuroimmunol.

[35] Corcione A, Casazza S, Ferretti E, Giunti D, Zappia E, Pistorio A (2004). Recapitulation of B cell differentiation in the central nervous system of patients with multiple sclerosis. Proc Natl Acad Sci U S A.

[36] Kornek B, Lassmann H (1999). Axonal pathology in multiple sclerosis. A historical note. Brain Pathol.

[37] Hesselgesser J, Taub D, Baskar P, Greenberg M, Hoxie J, Kolson DL (1998). Neuronal apoptosis induced by HIV-1 gp120 and the chemokine SDF-1 alpha is mediated by the chemokine receptor CXCR4. Curr Biol.

[38] Gardner C, Magliozzi R, Durrenberger PF, Howell OW, Rundle J, Reynolds R (2013). Cortical grey matter demyelination can be induced by elevated pro-inflammatory cytokines in the subarachnoid space of MOG-immunized rats. Brain.

[39] Vogel DYS, Vereyken EJF, Glim JE, Heijnen PDAM, Moeton M, van der Valk P (2013). Macrophages in inflammatory multiple sclerosis lesions have an intermediate activation status. J Neuroinflammation.

[40] Zhang Z, Zhang Z-Y, Schittenhelm J, Wu Y, Meyermann R, Schluesener HJ (2011). Parenchymal accumulation of CD163+ macrophages/microglia in multiple sclerosis brains. J Neuroimmunol.

[41] Fabriek BO, Møller HJ, Vloet RPM, van Winsen LM, Hanemaaijer R, Teunissen CE (2007). Proteolytic shedding of the macrophage scavenger receptor CD163 in multiple sclerosis. J Neuroimmunol.

[42] Boven LA, Van Meurs M, Van Zwam M, Wierenga-Wolf A, Hintzen RQ, Boot RG (2005). Myelin-laden macrophages are anti-inflammatory, consistent with foam cells in multiple sclerosis. Brain.

[43] Stilund M, Reuschlein A-K, Christensen T, Møller HJ, Rasmussen PV, Petersen T (2014). Soluble CD163 as a marker of macrophage activity in newly diagnosed patients with multiple sclerosis. PLoS One.

[44] Stilund M, Gjelstrup MC, Petersen T, Møller HJ, Rasmussen PV, Christensen T (2015). Biomarkers of inflammation and axonal degeneration/damage in patients with newly diagnosed multiple sclerosis: contributions of the soluble CD163 CSF/serum ratio to a biomarker panel. PLoS One.

[45] Komori M, Blake A, Greenwood M, Lin YC, Kosa P, Ghazali D (2015). Cerebrospinal fluid markers reveal intrathecal inflammation in progressive multiple sclerosis. Ann Neurol.

[46] Choi SR, Howell OW, Carassiti D, Magliozzi R, Gveric D, Muraro PA (2012). Meningeal inflammation plays a role in the pathology of primary progressive multiple sclerosis. Brain.

[47] Lucchinetti CF, Popescu BFGG, Bunyan RF, Moll NM, Roemer SF, Lassmann H (2011). Inflammatory cortical demyelination in early multiple sclerosis. N Engl J Med.

[48] Absinta M, Vuolo L, Rao A, Nair G, Sati P, Cortese ICM, et al. Gadolinium-based MRI characterization of leptomeningeal inflammation in multiple sclerosis.Neurology. 2015;85(1):18–28. 10.1212/WNL.0000000000001587 PMC4501940 25888557

