# Supplementary material for: Increased cortical lesion load and intrathecal inflammation is associated with oligoclonal bands in multiple sclerosis patients: a combined CSF and MRI study
Source: J Neuroinflammation. 2017 Feb 21;14:40. doi: 10.1186/s12974-017-0812-y (PMC5319028; doi:10.1186/s12974-017-0812-y)
Supplement: Additional file 1: — Diagram of the studied populations. (PPTX 61 kb) [file 12974_2017_812_MOESM1_ESM.pptx]

## Slide 1
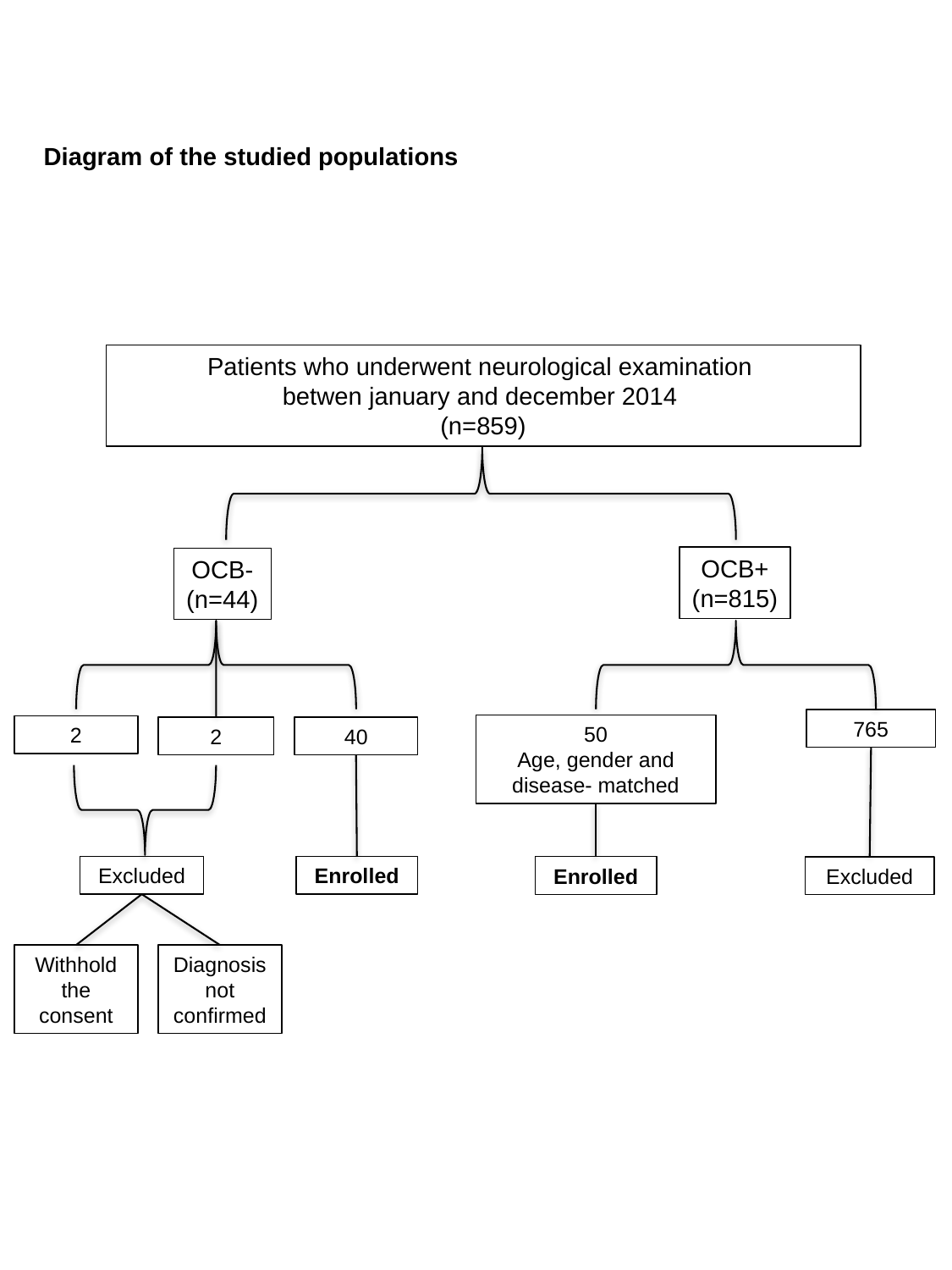

Diagram of the studied populations
Patients who underwent neurological examination
betwen january and december 2014
(n=859)
OCB+
(n=815)
OCB-
(n=44)
765
50
Age, gender and disease- matched
2
2
40
Excluded
Enrolled
Enrolled
Excluded
Withhold the consent
Diagnosis not confirmed
